# Molecular Dissection of the *SlBAG9* Promoter from Tomato and Its Thermo-Regulatory Activity

**DOI:** 10.3390/ijms27167496

**Published:** 2026-08-21

**Authors:** Fan Fei, Fan Yang, Yucheng Peng, Menghan Zhu, Sihan Li, Hailong Jiang, Haidong Ding

**Affiliations:** 1 College of Bioscience and Biotechnology, Yangzhou University, Yangzhou 225009, China; 2 College of Landscape and Horticulture, Yangzhou University, Yangzhou 225009, China

**Keywords:** tomato, *SlBAG9*, promoter, functional identification, high temperature

## Abstract

The Bcl-2-associated athanogene (BAG) gene family plays vital roles in plant growth, development, and biotic and abiotic stress responses. Previous work has demonstrated that tomato *SlBAG9*, a group II BAG member, negatively regulates plant thermotolerance. However, the regulatory mechanisms governing *SlBAG9* expression remain poorly understood. In this study, we isolated and characterized the authentic 1486 bp full-length promoter (P1) of *SlBAG9* from the tomato genome. Building upon our previous transcript-level observations, we provide here a detailed functional characterization of this promoter at the cellular and tissue level. In silico analysis identified several key *cis*-acting regulatory elements, including abscisic acid-responsive elements (ABRE), anaerobic response elements (ARE), and a heat shock element (HSE1). We used stable transgenic tomato plants carrying *SlBAG9pro::GUS* to verify that the full-length promoter was capable of driving the expression of β-glucuronidase reporter gene (GUS) in transgenic tomato plants, showing GUS staining was detectable in the roots, stems, leaves, flowers, fruits, and seeds, with the highest activity in red-ripe fruits. Notably, GUS activity was significantly upregulated by high temperature (HT) but not by PEG, NaCl, ABA, or cold treatments. To further dissect the HT-responsive regulatory module, we generated three 5′-terminal deletion fragments (−386 bp, P2; −239 bp, P3; and −113 bp, P4) and fused them to GUS. Under HT stress, the smallest deletion P4 showed negligible GUS activity, whereas P1, P2, and P3 retained significant activity. Furthermore, site-directed deletion of the HSE1 element in the full-length context (MU-P1) abolished HT inducibility, confirming that HSE1 serves as a critical positive HT-responsive element. Collectively, these findings confirm and extend our observations that *SlBAG9* is a stress-responsive gene, and the characterized HSE1-dependent promoter module represents a promising candidate for genetic engineering aimed at enhancing thermotolerance in crops.

## 1. Introduction

Transcriptional regulation is a central checkpoint linking complex gene expression networks to multi-layered developmental and stress responses [1]. The promoter is a key DNA sequence in gene expression, that directs RNA polymerase, which binds template DNA precisely, involved in regulating the expression patterns of specific genes [2]. Within promoters, diverse *cis*-acting elements serve distinct functions and play pivotal regulatory roles in gene expression [3], often through interactions with specific transcription factors (TFs) or other proteins [4]. Promoters can be classified into constitutive, inducible, tissue-specific, and synthetic types. Constitutive promoters, such as the cauliflower mosaic virus 35S promoter (CaMV35S) [5], drive high-level transgene expression across all plant tissues and have been widely employed in plant genetic engineering [6,7]. However, constitutive promoters drive high-level transgene expression across all tissues, which often leads to metabolic burden, energy waste, undesirable phenotypes, and abnormal growth [6,8,9,10].

Inducible and tissue-specific promoters are more significant as they can drive gene expression under specific conditions or in a tissue-specific manner; therefore, adverse effects caused by constitutive expression are avoided [8,11]. Such promoters are increasingly favored for precision plant improvement via transgenic approaches [6,7] and can effectively reduce unnecessary metabolic costs [7,11]. For instance, the pericarp-abundant promoter *AhGLP17-1P* was identified in peanut. Inducible promoters can respond to endogenous hormones, external chemical signals, and various stresses [8]. Under various stresses, inducible promoters ensure rapid and synchronized responses, including remodeling chromatin rapidly and combining with regulatory complexes, and this rapid response is essential for responding to damage caused by various stresses [12]. The soybean *GmRIQ2* promoter could be induced by IAA, ABA, MeJA, and light [7]; the rice *OsRGLP2* promoter was induced by drought, SA, NaCl, H_2_O_2_, and auxin [13]; and numerous others from carrot, banana, rice, grape, and maize [6,12,14,15,16]. Dave et al. [8] showed that the promoter of *AlHKT2;1* was involved in the response to abiotic stress and deletion of D2 (a 620 bp length deletion fragment at the 5′ end of the promoter) was sufficient to drive stress-induced gene expression to provide stress tolerance in plants, offering a potential target for engineering stress tolerance. Huo et al. [17] analyzed the promoters of the miltiradiene synthase (MS) gene *TwTPS27a/b* and found that TGACG-motif, a MeJA-responsive element, interacted with a MeJA-inducible TwTGA1, which was involved in the biosynthesis of triptolide induced by JA. Thus, a comprehensive understanding of promoter architecture and activity is fundamental to deciphering gene regulatory mechanisms and is a prerequisite for effective transgenic breeding.

BAG proteins are evolutionarily conserved molecular chaperone cofactors involved in diverse cellular processes, including proliferation, programmed cell death, and stress responses [18,19,20]. In plants, BAG family members are widely implicated in responses to high temperature (HT), drought, and salinity. For instance, overexpression of *AtBAG4* confers enhanced tolerance to cold, salt, oxidative stress, and UV light [18,19], while *AtBAG6* contributes to thermotolerance [21,22]. Overexpression of *TaBAG* and *TaBAG2* in Arabidopsis increases salt tolerance [23]. In rice, *OsBAG* genes are responsive to nutrient deficiency, HT, and drought, with *OsBAG1* and *OsBAG3* strongly induced by HT, and *OsBAG6* upregulated in drought-tolerant lines under water deficit [24]. Additional BAG genes have been characterized in soybean, tomato, banana, and grape [25,26,27,28,29]. Nawkar et al. [21] identified conserved stress-related *cis*-elements, including MYC, ABRE, HSE, DRE, and MYB binding sites, in the promoters of Arabidopsis BAG genes, suggesting their involvement in adaptive stress responses. Our previous studies found that there were a variety of elements connected with hormones and environmental stresses, for instance, ABA-responsive *cis*-elements, SA-responsive *cis*-elements, W-box, DRE, MBS, MYC and HSE in the promoters of ten *SlBAGs* [27]. However, direct experimental evidence linking plant BAG promoters to abiotic stress signaling remains limited.

In our previous studies, we found that tomato *SlBAG9* could respond to various stresses, such as HT, salt, drought, cold, and ABA [26,27], and that its overexpression confers hypersensitivity to HT and osmotic stress. In Ding et al. [26], we used two 5′-deletion constructs in transient tobacco assays to suggest that the HSE1 motif might be important for HT response. Jiang et al. [27] reported the organ-specific expression of *SlBAG9* by qRT-PCR. However, these earlier works did not provide definitive proof of the core HT-responsive element, nor did they establish the authentic promoter sequence or its cell-specific activity in stable transgenic tomato plants. In this study, we systematically characterized the upstream promoter of *SlBAG9* with three specific objectives: (1) to confirm the authentic promoter sequence and clarify the spatial and temporal distribution of *SlBAG9* activity by GUS histochemical staining in stable transgenic tomato; (2) to evaluate the promoter’s responsiveness to different stresses using quantitative fluorometric assays; and (3) to delineate the core HT-responsive element through fine 5′-terminal deletion and site-directed mutagenesis of the HSE1 motif, validated in both transient tobacco and stable tomato transformants. Together with our earlier transcript-level analyses, these findings provide a more detailed understanding of the transcriptional regulation governing *SlBAG9* as a negative regulator of thermotolerance and identify a promising regulatory module that could, in future studies, be leveraged for enhancing plant stress resilience through gene editing technologies (GETs).

## 2. Results

### 2.1. Cloning and Sequence of SlBAG9 Promoter

In a previous genome-wide study, we identified ten *SlBAG* genes and predicted that the 1500 bp region upstream of the *SlBAG9* translation start codon (ATG) contained core promoter elements and various *cis*-regulatory motifs associated with growth, hormone signaling, abiotic stress, and light responses, as determined by PlantCARE [27]. To further investigate *SlBAG9* function, we cloned its promoter to assess activity and regulatory mechanisms. A ~1500 bp fragment was amplified from wild-type tomato (*Solanum lycopersicum* ‘cv. Ailsa Craig’) genomic DNA using primers designed against the ITAG4.0 database (Supplementary Table S1). However, the actual length is 1486 bp after sequencing. To further verify the confidence level of the sequence, we carried out sequence alignment in the NCBI database. Sequencing results demonstrated that our cloned fragment was completely identical to the deposited sequence database OU640353 and shared 99% identity with database HG975522. However, notable divergence was observed when compared to the ITAG4.0 reference (98% identity) (Supplementary Figure S1). Because the ITAG genome data is from tomato Heinz 1706, we further cloned and sequenced the promoter from Heinz 1706 DNA, obtaining identical results to those from Ailsa Craig. Therefore, we confirmed that the authentic *SlBAG9* promoter is 1486 bp in length.

### 2.2. cis-Element Analysis of SlBAG9 Promoter

The 1486 bp promoter sequence was submitted to the PlantCARE database (https://www.plantcare.co.uk/) for *cis*-acting regulatory element analysis. The analysis was performed using the default parameters of the PlantCARE tool, which identifies putative regulatory elements based on a comprehensive plant *cis*-element database. Re-analysis confirmed the presence of core TATA-box and CAAT-box motifs, along with a variety of stress-related *cis*-elements, including ABRE and AAGAA-motif (ABA responsiveness), ARE (anaerobic induction), MYB and MYC binding sites, MBS (drought-inducible MYB-binding site), LTR (low-temperature responsiveness), and HSE1 (heat shock). Additional light-responsive elements, the zein metabolism regulator O2-site, and several uncharacterized motifs were also identified. The major stress-associated elements are summarized in Supplementary Table S2.

### 2.3. Construction of SlBAG9 Promoter::GUS Plasmid

**Figure 1:**
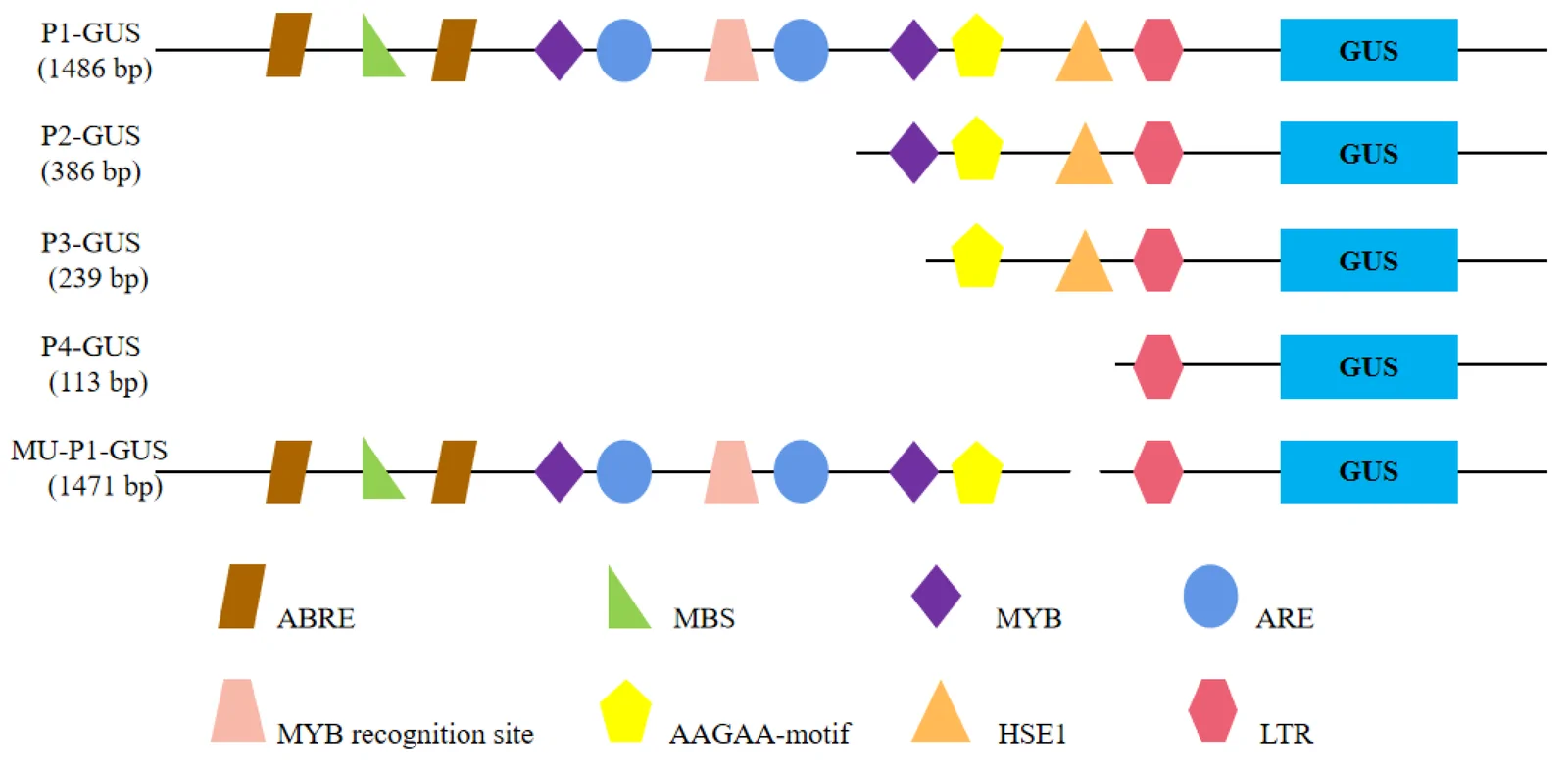
Construction of recombinant expression vectors for *SlBAG9* promoter::GUS. Only *cis*-acting elements in response to stresses are shown. P1: full-length promoter of *SlBAG9* gene, P2: a deletion fragment at the 5′ end of 386 bp in length, P3: a deletion fragment at the 5′ end of 239 bp in length, P4: a deletion fragment at the 5′ end of 113 bp in length (without HSE1 element), MU-P1: the promoter full-length fragment with site-directed deletion of HSE1 element.

Promoter-specific primers (P1F, P2F, P3F, P4F, and PR) were used to amplify the full-length 1486 bp promoter and three 5′-deletion fragments (−386 bp, −239 bp, and −113 bp, referring to the lengths of the remaining promoter fragments upstream of the ATG) from tomato genomic DNA. These fragments were cloned into the pBI121 vector upstream of the *GUS* reporter gene, replacing the CaMV35S promoter, to generate P1-GUS, P2-GUS, P3-GUS, and P4-GUS, respectively (Figure 1). To assess the specific role of HSE1, a full-length promoter with a site-directed internal deletion of the HSE1 motif (MU-P1-GUS) was also constructed. All promoter::*GUS* constructs were initially tested via *Agrobacterium*-mediated transient expression in tobacco leaves. For stable transformation, P1-GUS, P4-GUS, and MU-P1-GUS were introduced into tomato.

### 2.4. Histochemical Localization of SlBAG9 Promoter Activity in Transgenic Tomato

**Figure 2:**
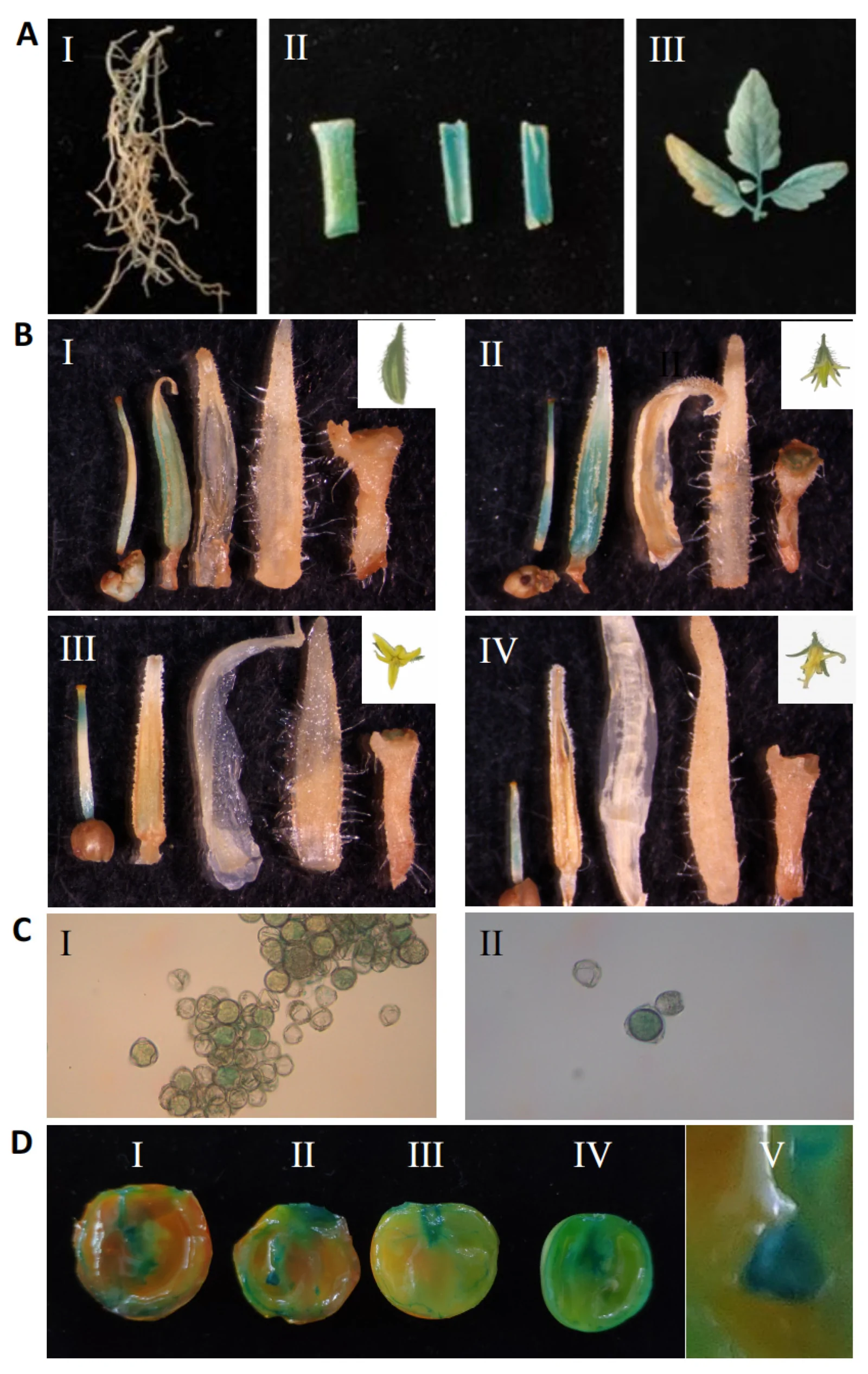
Histochemical localization of GUS activity in different organs and tissues. (**A**) I, roots, II, stems, and III, leaves; (**B**) flowers at different times, I, flower bud stage (approximately 5 days before anthesis); II, pre-anthesis stage (approximately 1 days before anthesis); III, full open stage (anthesis, 0 DPA); IV, flowering waning period (3 DPA). DPA, days post-anthesis; (**C**) pollen grain, I is clustered, II is single; (**D**) fruits and seeds, I is red ripening stage, II is firm ripening stage, III is color turning stage, IV is green ripening stage, V is seeds.

To determine the spatiotemporal expression pattern of *SlBAG9*, GUS histochemical staining was performed on various tissues of P1-GUS transgenic plants. GUS activity was detected in roots, stems, leaves, flowers, fruits, and seeds (Figure 2). The strongest staining was observed in red-ripe fruits, followed by flowers, with notable expression also in seeds. This pattern is largely consistent with the organ-specific expression profile of *SlBAG9* determined by qRT-PCR in our earlier study [27]. Within flowers, GUS staining was predominantly localized to the pistils and stamens, with little or no signal in petals, calyx, or receptacle. As flowers developed, GUS expression in stamens gradually diminished, whereas pistil staining intensified (Figure 2B). Pollen grains also showed clear GUS activity (Figure 2C). Across fruit developmental stages, GUS staining was detectable at all stages but increased markedly during ripening (Figure 2D). These results suggest that *SlBAG9* may participate in vegetative and reproductive growth regulation in tomato. Notably, this cell-specific spatial mapping—including detection in pollen grains and distinct pistil/stamen patterns—was not previously available from qRT-PCR data [27] and represents a new contribution of this study.

### 2.5. Stress Responsiveness of the SlBAG9 Promoter in Transgenic Tomato

**Figure 3:**
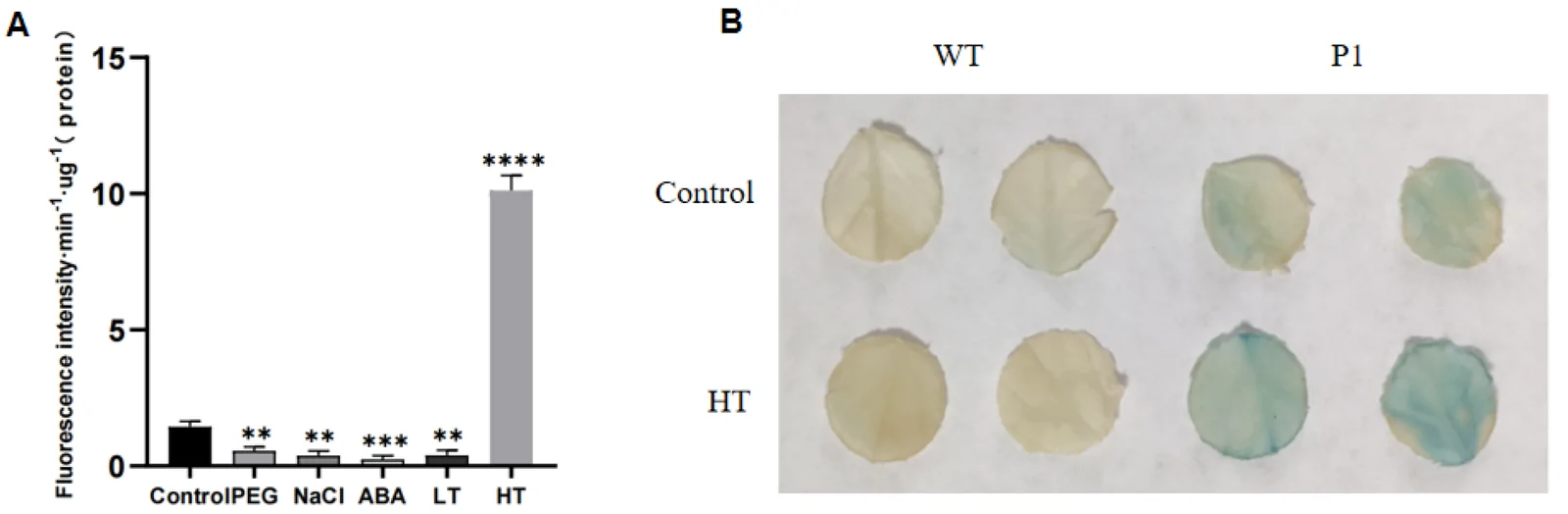
GUS activity of P1 under different stresses. (**A**) Fluorescence quantitative analysis of GUS enzyme activity; (**B**) GUS histochemical staining. Control: with no treatment, PEG: treatment of 15% PEG, NaCl: treatment of 175 mM salt, ABA: treatment of 100 μM ABA, LT: treatment of cold (4 °C for 6 h), HT: treatment of high temperature (42 °C for 6 h). The data are shown as the mean ± SE (*n* = 3), with significant differences revealed by one-way ANOVA with Dunnett’s test (** for *p* < 0.01, *** for *p* < 0.001, **** for *p* < 0.0001). (**B**) Results of GUS staining under high-temperature stress. WT: wild-type tomato plant, P1: full-length promoter of the *SlBAG9* transgenic tomato plant.

To evaluate the response of the *SlBAG9* promoter to different stresses, P1-GUS transgenic tomato plants were subjected to 6 h treatments with drought (PEG), salt (NaCl), HT, low temperature (LT), and ABA. GUS enzymatic activity (using MUG as substrate) was significantly reduced under drought, salt, cold, and ABA, but markedly increased under HT (Figure 3A). GUS histochemical staining confirmed the HT-induced upregulation (Figure 3B). These results align with the stress-responsive expression pattern of *SlBAG9* previously reported by our group [27], reinforcing the conclusion that *SlBAG9* is strongly and specifically induced by high temperature.

### 2.6. HSE1 Is the Core Element of SlBAG9 Promoter Responding to HT

**Figure 4:**
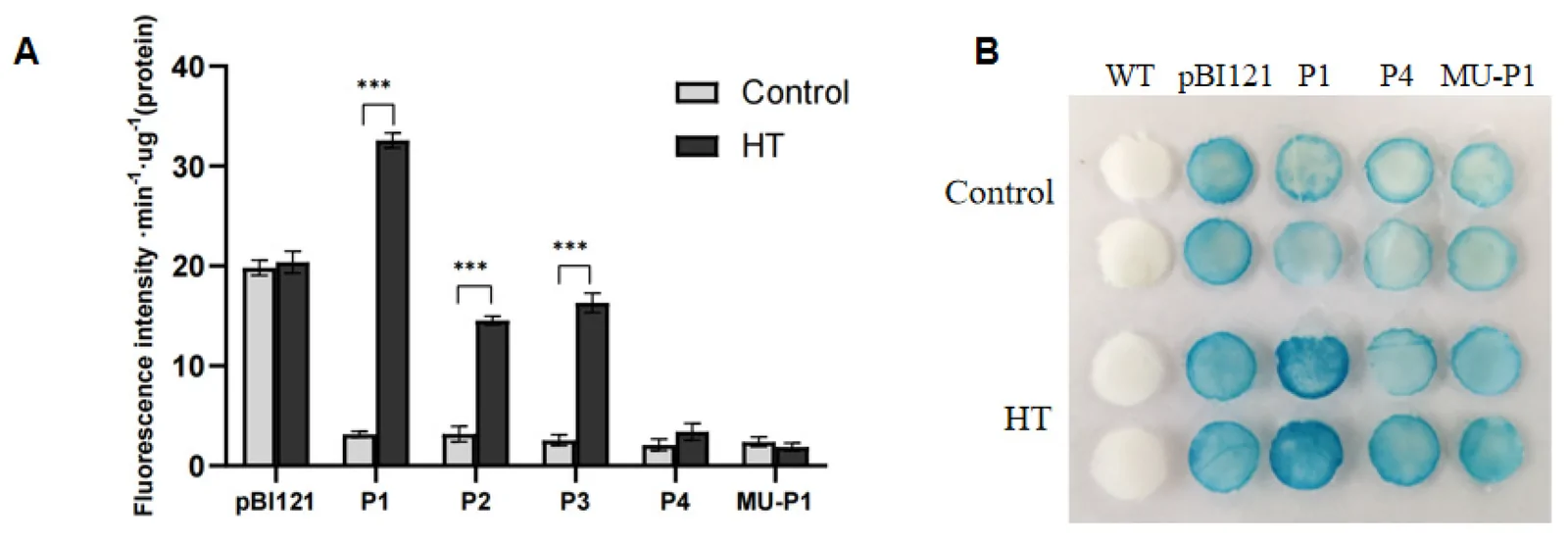
Transient expression of GUS activity in tobacco after high-temperature stress. (**A**) Fluorescence quantitative analysis of GUS enzyme activity; (**B**) GUS histochemical staining. Control: with no treatment, HT: treatment of high temperature (42 °C for 6 h), WT, wild-type plants; pBI121, plants constitutively expressing GUS driven by the CaMV35S promoter (positive control); P1, plants expressing GUS driven by the full-length (1486 bp) *SlBAG9* promoter; P2, plants expressing GUS driven by the 5′-terminal deletion fragment from −386 bp to the ATG; P3, plants expressing GUS driven by the 5′-terminal deletion fragment from −239 bp to the ATG; P4, plants expressing GUS driven by the 5′-terminal deletion fragment from −113 bp to the ATG, which lacks the HSE1 element; MU-P1, plants expressing GUS driven by the full-length promoter carrying a site-directed internal deletion of the HSE1 motif. The data are shown as the mean ± SE (*n* = 3), with significant differences revealed by one-way ANOVA with Student’s *t* tests (*** for *p* < 0.001).

To further explore the core elements of *SlBAG9* promoter responding to HT, the three 5′-deletion constructs (P2, P3, and P4) were transiently expressed in tobacco leaves, followed by HT treatment and quantitative GUS activity measurement. Both P2 and P3, which retain the HSE1 element, showed significant HT-inducible GUS activity comparable to full-length P1. In contrast, P4, which lacks HSE1, exhibited no HT induction (Figure 4A). To further verify the function of HSE1 element responding to HT, the MU-P1 construct (full-length promoter with a specific HSE1 deletion) was tested. Unlike P1, MU-P1 completely lost HT-inducible GUS activity (Figure 4B), which was the same as the results of P4.

**Figure 5:**
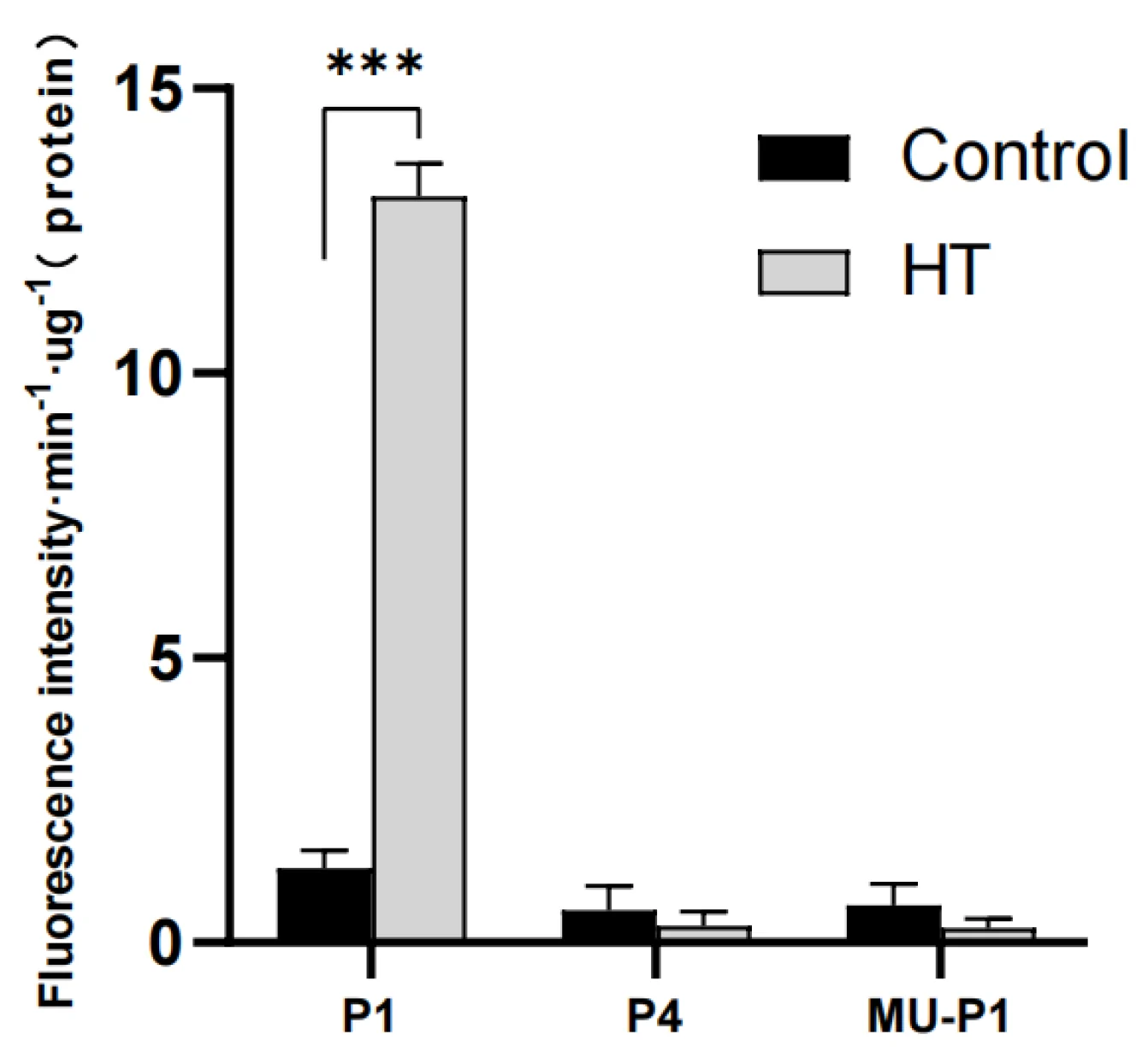
GUS activity in transgenic tomato plants after high-temperature treatment. Control: no treatment; HT: treatment of high temperature (42 °C for 6 h); P1, transgenic plants harboring the full-length (1486 bp) *SlBAG9* promoter::GUS construct; P4, transgenic plants harboring the 5′-terminal deletion construct (−113 bp, lacking HSE1)::GUS; MU-P1, transgenic plants harboring the full-length promoter with a site-directed internal deletion of the HSE1 motif::GUS. The data are shown as the mean ± SE (*n* = 3), with significant differences revealed by one-way ANOVA with Student’s *t* tests (*** for *p* < 0.001).

In addition to the verification of tobacco transient expression, we also transformed P1, P4, and MU-P1 recombinant plasmids into tomato and obtained transgenic plants to verify the central role of HSE1 element in response to HT. After HT treatment, GUS activity increased significantly in P1 plants, while P4 and MU-P1 lines showed no significant change (Figure 5). Unlike our previous study [26], which relied solely on transient tobacco assays with histochemical staining, here we provide quantitative fluorometric data from stable transgenic tomato plants—representing the gold standard for promoter analysis. Collectively, the findings indicated that HSE1 is the core and indispensable element of *SlBAG9* promoter responding to HT.

## 3. Discussion

In plants, BAG family proteins participate in a wide range of biological processes, including programmed cell death and autophagy, and exhibit functional conservation in stress responses. Recent studies have increasingly focused on the regulatory roles of BAGs [20]. For example, Arabidopsis WRKY29 positively regulates *AtBAG7* expression by binding to the W-box motif in its promoter, thereby enhancing thermotolerance [30]. In our earlier work, we found that tomato *SlBAG9* responds to multiple stresses, including HT, salt, drought, cold, and ABA [26,27], and that its overexpression compromises thermotolerance. Promoters are key DNA sequences that direct RNA polymerase binding and regulate downstream gene expression [31]; thus, dissecting promoter function is essential for understanding the regulatory logic of target genes. We therefore hypothesized that the *SlBAG9* promoter plays a pivotal role in HT signaling and set out to characterize it.

The GUS reporter system is widely used for promoter analysis, enabling both histochemical localization and quantitative fluorometric assays [7,32]. Although we previously profiled *BAG* gene expression across organs and under stress conditions at the transcript level [27], direct evidence of promoter activity and its regulatory mechanisms was lacking. In the present study, GUS fusions revealed that the *SlBAG9* promoter drives expression in roots, stems, leaves, flowers, fruits, and seeds (Figure 2). Notably, within flowers, GUS activity was concentrated in pistils and stamens, with staminal expression declining during flower maturation (Figure 2B). Pollen grains also exhibited GUS staining (Figure 2C). We speculate that the decline in staminal staining may reflect pollen release, while increasing pistil expression could be linked to pollination. Moreover, the progressive increase in GUS activity during fruit ripening (Figure 2D) supports a role for *SlBAG9* in developmental regulation [20]. The high promoter activity during fruit ripening (Figure 2D) may reflect a dual role: supporting developmental processes and priming the fruit for stress responses that often accompany ripening. Since fruits are highly sensitive to environmental stress during ripening, the promoter’s HT responsiveness at this stage could have evolved to fine-tune stress and developmental signals. The cellular resolution provided by histochemical staining offers new insights that were not accessible through transcriptional profiling alone. These spatial details lay the groundwork for future investigations into the specific developmental functions of *SlBAG9*, which remain to be elucidated.

Analysis of *cis*-acting regulatory elements in the gene promoter regions is useful for deciphering the molecular mechanism governing *SlBAG9* gene expression in response to stresses [17]. In this study, several abiotic stress-responsive *cis*-acting elements were identified in the promoter of *SlBAG9*, including ABRE, MBS, MYB, and MYC binding sites, as well as HSE1. MYBs are found to regulate the tolerance of plant under salt and drought stress and ABRE, MYB, and MYC binding sites might also be helpful for drought and salt response [33]. In our GUS activity assays, the *SlBAG9* promoter was repressed by drought, salt, cold, and ABA, but strongly induced by HT (Figure 3A), which closely mirrors the expression pattern of the endogenous gene [27]. Although the negative regulation under non-HT stresses remains to be mechanistically confirmed, these elements are candidates for further verification. Here, the HSE1 element with HT response attracted our particular attention.

The 5′-deletion approach is a well-established strategy for mapping regulatory elements within promoters. For instance, Chen et al. [6] used 5′-deletions to functionally dissect the rice *OsHAK1* promoter under osmotic/drought stress, and Tounsi et al. [34] applied similar deletions to the *TmHKT1;4-A2* promoter to study abiotic stress responses. In our previous study, we found that *SlBAG9* was a Bcl-2 related anti-apoptotic gene in tomato and can respond to HT. The core element of *SlBAG9* promoter in response to HT is between −389 bp and −113 bp [26] and it was speculated that the HSE1 element in the promoter of *SlBAG9* might be involved in HT response, which was supported by other studies [35,36]. In order to further clarify the core region of *SlBAG9* promoter responsive to HT, we constructed a 239 bp fragment (P3) and demonstrated that the key region is between −239 bp and −113 bp (Figure 4A). The HSE1 motif (tGAAgcTTCtgGAAt) is a canonical transcription factor binding site activated by HT [37]. Notably, the Arabidopsis ortholog *AtBAG5* is also HT-induced in stems and roots and contains HSE1 in its promoter, though its function had not been tested [21]. Our deletion analysis showed that only fragments retaining HSE1 responded to HT, while P4, which lacks HSE1, lost inducibility. This provides the first direct evidence that HSE1 is the core HT-responsive element in the *SlBAG9* promoter.

Site-directed mutagenesis using overlap extension PCR is an essential technique to introduce defined point mutations into target DNA sequences. It is not only beneficial for the generation of new genes, but also allows the analysis of gene fragments and protein function [38]. To clarify the central role of HSE1 element in the response of *SlBAG9* promoter to HT, we constructed a full-length promoter recombinant plasmid with site-directed deletion of the HSE1 element and transiently transformed it into tobacco leaves and constructed transgenic tomato plants for GUS activity analysis. The results in both tobacco and tomato leaves showed that the fragment without the HSE1 element (P4-GUS and MU-P1-GUS) did not change significantly after HT treatment (Figure 4 and Figure 5). These findings unequivocally establish that the HSE1 element is the core element of the promoter of *SlBAG9* responding to HT, and the expression of *SlBAG9* is positively regulated under HT. While our data establish HSE1 as the essential and non-replaceable core element for the HT response, the possibility that additional, yet-unidentified *cis*-elements contribute to the full magnitude of induction cannot be excluded.

The apparent paradox of HT-inducible *SlBAG9* expression and its negative role in thermotolerance raise an intriguing question. It is tempting to speculate that this reflects a strategy to fine-tune the heat stress response (HSR). SlBAG9 possesses a conserved BAG domain (BD) that interacts with the ATPase domain of Hsp70/Hsc70 chaperones, and a plant-specific IQ calmodulin (CaM)-binding motif. A plausible hypothesis, which remains to be tested, is that upon HT-induced overaccumulation, SlBAG9 may “hijack” the Hsp70/Hsc70 chaperone system, disrupting its role in refolding or clearing denatured proteins, thereby exacerbating heat-induced proteotoxicity. Alternatively, through its IQ motif, SlBAG9 might interfere with Ca^2+^-mediated signaling pathways critical for thermotolerance. Based on these considerations, we propose the hypothesis that SlBAG9 functions as a negative feedback regulator, preventing an overactive and metabolically costly HSR and facilitating a timely return to growth after stress recovery [22,33]. Testing this hypothesis will require future investigations, direct evidence, such as protein–protein interaction or downstream marker assays.

## 4. Materials and Methods

### 4.1. Plant Materials and Growth Conditions

Tomato (*Solanum lycopersicum* ‘cv. Ailsa Craig’) wild-type and transgenic lines harboring promoter::GUS constructs were grown in small pots (8 × 8 cm) containing nutrient soil in a greenhouse at 25 °C/20 °C (day/night), 16 h light/8 h dark, and 70–75% relative humidity (RH). Plants were further treated at the stage of 5-leaf.

*Nicotiana benthamiana* seeds were evenly scattered on the nutrient soil, covered with plastic wrap to keep moist, and germinated for about 7 days. The germinated seedlings were moved into pots with nutrient soil and grown in a greenhouse under 25 °C/20 °C (day/night), 16 h light/8 h dark, and 70% RH. The plants were cultured for about 1 month for the transient expression experiment.

### 4.2. Promoters Sequence Analysis and SlBAG9 Promoter::GUS Expression Vectors Construct

**Figure 6:**
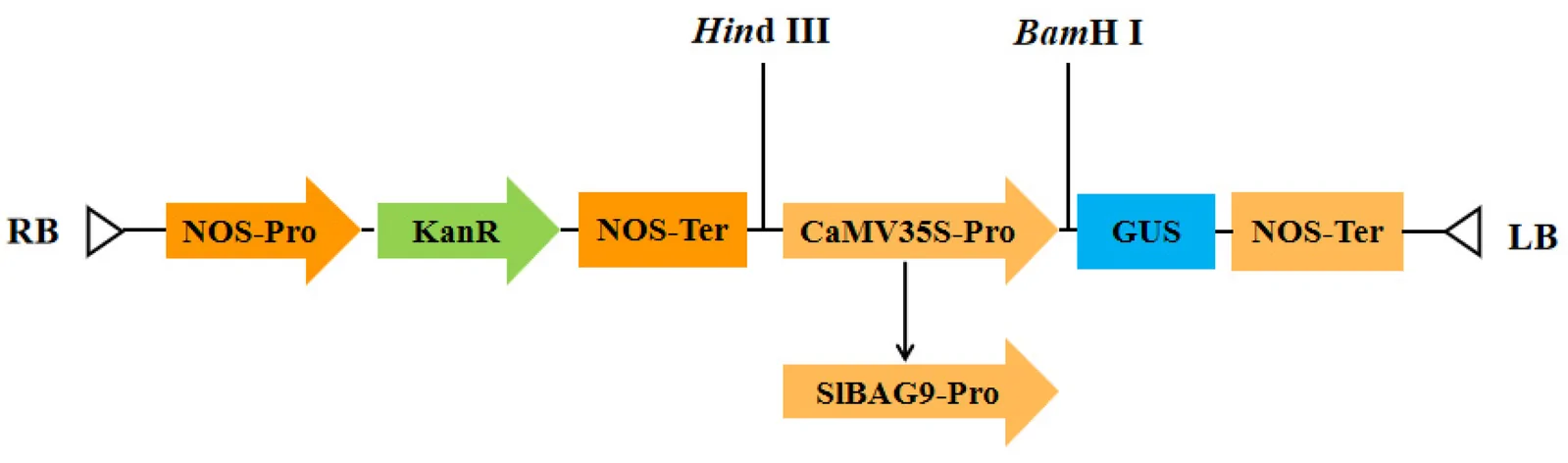
Schematic diagram of the construction of *SlBAG9* promoter::*GUS*.

To validate the promoter-driven activity, a 1500 bp promoter upstream of the *SlBAG9* gene (1500 bp upstream of ATG) was retrieved from Tomato ITAG4.0 database (Sol Genomics Network). According to the 1500 bp promoter sequence, we designed specific primers to amplify the promoter of *SlBAG9* from genomic DNA of wild-type tomato (*Solanum lycopersicum* ‘cv. Ailsa Craig’). The homologous recombinant primers were designed according to the sequences obtained, respectively named as P1-F and P-R (Supplementary Table S1). The cycling conditions for RT-PCR were as follows: 95 °C for 3 min; 35 cycles of 95 °C for 15 s, 58 °C for 15 s; 72 °C for 90 s; 72 °C for 5 min. The plant expression vector pBI121 was double digested with *Bam*HI and *Hin*dIII. The PCR products were purified, recovered, and connected to vector pBI121 to replace the CaMV 35S promoter (Figure 6). The connecting products were sequenced and the actual sequence length was 1486 bp, regarded as the full-length promoter P1 and the online website PlantCARE (https://www.plantcare.co.uk/) was used for *cis*-acting element analysis. pBI121 CaMV35S::GUS was used as a positive control in subsequent transformation studies as the 35S::GUS expression cassette.

To locate the HT-responsive core region, three deletion fragments at the 5′ end of *SlBAG9* of different sizes (−386/P2, −239/P3, and −113 bp/P4; and the “A” in “ATG” of *SlBAG9* was appointed as 1) were amplified from 1486 bp promoter of *SlBAG9* using the sequence special primers (Supplementary Table S1) that were designed on the basis of the location of *cis*-acting elements. To further confirm the role of HSE1 element in response to HT, the site-directed deletion of HSE1 element of 1486 bp (MU-P1) was constructed by overlap extension PCR. The amplification primers are shown in Supplementary Table S1.

### 4.3. SlBAG9 Promoter::GUS Plasmids Transformation, Transient Expression and HT Treatment

All *SlBAG9* promoter::GUS recombinant plasmids (pBI121-P1, pBI121-P2, pBI121-P3, pBI121-P4, and pBI121-MU-P1) and pBI121 (CaMV35S::GUS, serving as the positive control) were introduced into *Agrobacterium tumefaciens* GV3101 and incubated at 28 °C for about 2 days; single colonies were selected for PCR detection. Positive bacteria were chosen and incubated in LB liquid medium with kanamycin and rifampicin at 28 °C for about 16 h. Then, the transient expression in tobacco leaves was performed based on the way of Yang et al. [39] with the following details: bacterial suspension at OD600 = 0.6–0.8 in infiltration buffer (10 mM MES-KOH pH 5.6, 10 mM MgCl_2_, 150 µM acetosyringone), infiltrated into fully expanded leaves of 4-week-old plants. About 36 h after instantaneous expression in tobacco leaves, plants in the control group were treated in a normal environment, while plants in the treatment group were treated at 42 °C for 6 h. After that, GUS histochemical staining and GUS enzyme activity fluorescence quantitative analysis were performed. Three biological replicates were set in the experiment.

### 4.4. Generation of Transgenic Tomato Plants

Tomato tissue culture was performed based on the method of Ding et al. [40]. The recombinant plasmids (pBI121-P1, pBI121-P4, and pBI121-MU-P1) were introduced into WT tomato (*Solanum lycopersicum* ‘cv. Ailsa Craig’) by agrobacterium-mediated transformation. The tissue culture seedlings with kanamycin resistance were further identified by PCR. The transgenic T2 plants were used for further experiments. For each construct, two independent T2 lines were analyzed.

To determine the spatiotemporal expression pattern of the *SlBAG9* promoter, various tissues were collected from P1-GUS transgenic tomato plants at different developmental stages. Roots, stems, and fully expanded third true leaves were harvested from 7-leaf-stage seedlings. Flowers at four distinct developmental stages were collected: I, flower bud stage (approximately 5 days before anthesis); II, pre-anthesis stage (approximately 1 day before anthesis); III, full open stage (anthesis, 0 DPA); and IV, flowering waning period (3 DPA). A total of 3-5 intact flowers were harvested at each stage. Fruits at four different maturity stages were also collected: I, red ripe stage; II, firm ripe stage; III, color turning stage; and IV, green ripening stage. A total of 3-5 fruits were harvested at each stage. All collected samples were immediately processed for GUS histochemical staining as described below.

To explore the response of the *SlBAG9* promoter to different abiotic stresses, 5-leaf-stage P1-GUS transgenic tomato plants were subjected to the following treatments for 6 h: drought stress was imposed by irrigating the plants with 15% (*w*/*v*) polyethylene glycol (PEG) 6000 solution; salt stress was applied by irrigating with 175 mM NaCl solution; ABA treatment was performed by spraying 100 μM abscisic acid (ABA) solution onto the leaves until run-off; low-temperature (LT) stress was applied by transferring the plants to a growth chamber set at 4 °C; and high-temperature (HT) stress was applied by transferring the plants to a growth chamber set at 42 °C. Untreated plants maintained at 25 °C served as the control. To confirm the HT response, P1, P4, and MU-P1 transgenic plants were treated at 42 °C for 6 h, with untreated plants as controls. Each treatment included three biological replicates.

### 4.5. GUS Histochemical Staining

GUS staining was performed according to Jefferson et al. [41]. In brief, about one-month-old tobacco leaves were transient expressed, rinsed with distilled water, immersed in GUS staining solution which including 10 mM EDTA (pH 8.0), 0.1% (*v*/*v*) Triton X-100, 0.5 mM K_4_Fe(CN)_6_, 0.5 mM K_3_Fe(CN)_6_, 1 × phosphate buffer (27 mM KCl, 100 mM Na_2_HPO_4_, 20 mM KH_2_PO_4_ and 1.37 M NaCl), and 0.5 mg/mL 5-bromo-4-chloro-3-indolyl-β-d-glucuronide (X-Gluc) and ddH_2_O, and allowed to stand overnight at 37 °C for staining in dark. Subsequently, tobacco leaves dabbed with a paper towel to dry the surface of GUS stain solution were decolorized with absolute ethanol until there was no green coloration, and observed.

To explore the spatial expression of the *SlBAG9* gene, roots, stems, leaves, flowers at different stages (flower bud period, full opening period, and flowering waning period) [42], fruits at different maturity stages (green ripening stage, color turning stage, firm ripening stage, red ripening stage), and seeds were collected from the mature plants for GUS histochemical staining.

### 4.6. Fluorometric GUS Activity Assay

GUS enzyme activity was quantified fluorometrically using 4-methylumbelliferyl-β-d-glucuronide (4-MUG) as substrate, following Jefferson et al. [43]. GUS enzyme extraction buffer which including 0.05 M phosphate buffer with PH of 7.0, 0.01 M EDTA with PH of 8.0, 0.1% SDS, 0.1% (*v*/*v*) Triton X-100, 0.1% (*v*/*v*) β-mercaptoethanol (BME), and ddH_2_O were used to extract GUS enzyme from tobacco leaves and tomato leaves. The GUS enzyme solution was saved at 4 °C until use. Protein concentration was detected by the Coomassie brilliant blue method at 595 nm in a UV spectrophotometer. Then, 100 μL protein supernatant was added to 400 μL GUS extraction buffer preheated at 37 °C, 500 μL 4-methy-lumbelliferyl-b-glucuronide (4-MUG) substrate was added, and the solution was incubated in a warm bath at 37 °C. At different time gradients, 200 μL of mixed reactants was added to 800 μL reaction termination solution (0.2 M Na_2_CO_3_), then the mixture in a dark environment was saved at normal temperature. Fluorescence was measured at 455 nm (excitation 365 nm) using a microplate reader (Spark, TECAN, Männedorf, Switzerland). GUS activity was expressed as fluorescence change per unit protein per unit time. Each experiment was performed in triplicate.

### 4.7. Statistical Analysis

Each experiment was independently repeated at least three times. Statistical processing was completed via one-way ANOVA followed by Dunnett’s test or Student’s *t* tests in SPSS 22.0, with all quantitative results expressed as mean ± SE. Asterisks are used to label different significance levels (* for *p* < 0.05, ** for *p* < 0.01, *** for *p* < 0.001, **** for *p* < 0.0001).

## 5. Conclusions

In this study, we isolated and functionally characterized the authentic 1486 bp promoter of tomato *SlBAG9*. Our results confirm that the full-length promoter directs GUS expression in roots, stems, leaves, flowers, fruits, and seeds, with distinct developmental dynamics in flowers and fruits, suggesting a role for *SlBAG9* in growth and development. We also demonstrate that the promoter is responsive to multiple stresses and hormones, with GUS activity significantly repressed by drought, salt, cold, and ABA, but strongly induced by HT. Furthermore, through systematic 5′-deletion and site-directed mutagenesis, we provide direct evidence confirming HSE1 as the core element mediating HT-induced expression. Taken together, this work solidifies our understanding of the transcriptional control of SlBAG9 and identifies its HSE1-dependent promoter module as a promising candidate for future applications in engineering stress resilience, pending functional validation through targeted mutagenesis or knockout studies.

## Data Availability

The original contributions presented in this study are included in the article/Supplementary Materials. Further inquiries can be directed to the corresponding authors.

## Supplementary Materials

The following supporting information can be downloaded at: https://www.mdpi.com/article/10.3390/ijms27167496/s1.
