# Supplementary material for: Molecular Dissection of the SlBAG9 Promoter from Tomato and Its Thermo-Regulatory Activity
Source: Int J Mol Sci. 2026 Aug 21;27(16):7496. doi: 10.3390/ijms27167496 (PMC13512930; doi:10.3390/ijms27167496)
Supplement: Supplementary file 1 [file ijms-27-07496-s001.zip › ijms-4492361-supplementary.pdf]

**Table S1.** The primer sequences.

| Primer name | Primer sequence (5' to 3') | Application |
|---|---|---|
| P1-F | **aagctt**CGCAGGAGCCCGCGATGTCAAA | full-length promoter clone forward primer |
| P2-F | **aagctt**AAAGATTTAGCCCACAGA | 5' -end deletion fragment 2 clone forward primers |
| P3-F | **aagctt**TGGCGAAAGAATTTGACT | 5' -end deletion fragment 3 clone forward primer |
| P4-F | **aagctt**TCGAGTCTCAATCTGGAAA | 5' -end deletion fragment 4 clone forward primers |
| P-R | **ggatcc**TGTTAACTTTGATCAGTTTTGATAGC | reverse primer |
| MU-P1-F | gccttccggaagatgttATTCGAGTCTCAATCTGGAAAATTC | deletion HSE1 element forward primers |
| MU-P1-R | aaacatcttccggaaggcATCTTAGCTTTTGCT | deletion HSE1 element reverse primers |

**Table S2.** Identification of major cis-acting elements associated with abiotic stress responses in the *SlBAG9* promoter region.

| Element | Sequence | Function | Number | Position |
|---|---|---|---|---|
| ABRE | ACGTG/CACGTG | cis-acting element involved in the abscisic acid responsiveness | 5 | 289(+), 290(+), 297(+), 534(+), 535(+) |
| MBS | CAACTG | MYB binding site involved in drought-inducibility | 1 | 424(-) |
| MYB | CAACCA | | 2 | 730(+), 1243(-) |
| ARE | AAACCA | cis-acting regulatory element essential for the anaerobic induction | 2 | 761(+), 1001(+) |
| MYB recognition site | CCGTTG | | 1 | 984(+) |
| AAGAA-motif | GAAAGAA | Cis-acting elements involved in abscisic acid responsiveness | 1 | 1251(+) |
| HSE1 | TGAAGCTTCTGGAAT | Cis-acting elements involved in high temperature reactions | 1 | 1356(+) |
| LTR | CCGAAA | cis-acting element involved in low-temperature responsiveness | 1 | 1415(+) |

Note: The 1486 bp promoter sequence was submitted to the PlantCARE database (https://www.plantcare.co.uk/)

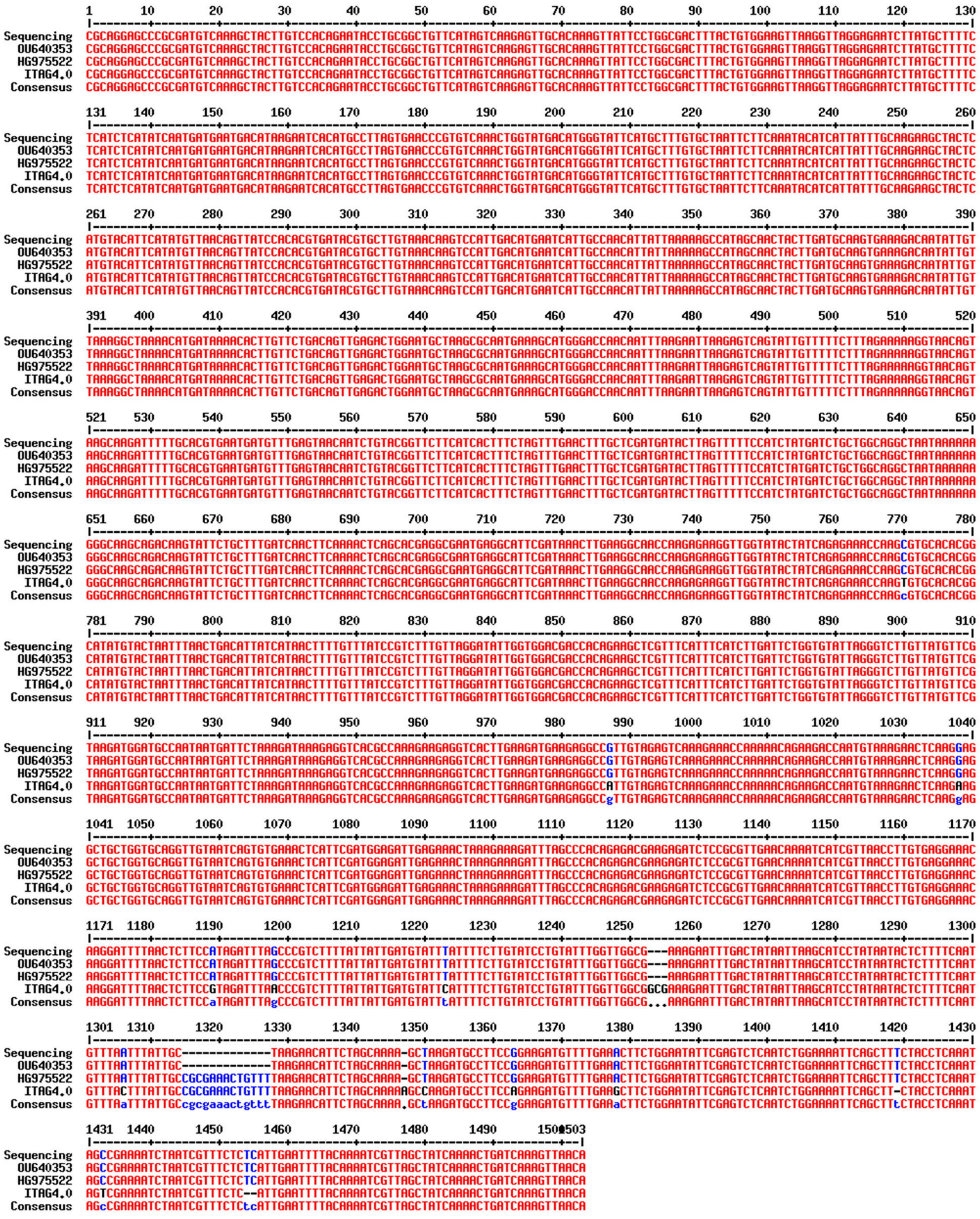


**Figure S1.** Sequence alignment of three databases of 1500 bp promoter upstream of *SlBAG9* gene. The actual sequencing sequence is used for alignment with OU640353, HG975522 and ITAG4.0 using online tool (http://multalin.toulouse.inra.fr/multalin/).
